# Metabotropic glutamate receptor 5 in the anterior cingulate cortex predicts individual differences in motor impulsivity but not in risky decision-making

**DOI:** 10.1038/s41398-026-03951-5

**Published:** 2026-03-23

**Authors:** Florian Marchessaux, Chloé Arrondeau, Raphaël Goutaudier, Ginna Urueña-Méndez, Nathalie Ginovart

**Affiliations:** 1 Department of Psychiatry, Faculty of Medicine, University of Geneva, Geneva, Switzerland; 2 Department of Basic Neurosciences, Faculty of Medicine, University of Geneva, Geneva, Switzerland

**Keywords:** Molecular neuroscience, Neuroscience

## Abstract

Impulsivity is a multidimensional construct implicated in various psychiatric disorders, yet its neurobiological underpinnings are insufficiently understood. The metabotropic glutamate receptor type 5 (mGluR5) has emerged as a promising target for modulating impulsive behavior, but whether impulsivity is associated with alterations in mGluR5 availability is unknown. Here, we investigated mGluR5 availability in relation to different impulsivity constructs in male Roman high- (RHA) and low-avoidance (RLA) rat lines, which exhibit divergent impulsive profiles. Motor impulsivity and risky decision-making were assessed using the rat gambling task prior to positron emission tomography measurement of brain mGluR5 availability. Compared to low-impulsive RLA rats, high-impulsive RHA rats showed reduced mGluR5 availability across multiple brain regions. Voxel-wise analysis localized these deficits primarily in the motor cortex, anterior cingulate cortex (ACC), and mediodorsal thalamus. Voxel-wise analyses further highlighted a strong inverse relationship between mGluR5 availability in the ACC and premature responding, but not risky-decision making. Region-of-interest analysis showed that the ACC exhibited a robust within-line association between mGluR5 deficits and premature responses in low-impulsive RLA rats, along with a near-significant relationship in high-impulsive RHA rats. No other brain region demonstrated such consistent within-line correlations across both phenotypes. These findings provide the first evidence that localized mGluR5 reductions predict individual differences in motor impulsivity, with the ACC emerging as the region most consistently associated with this relationship. Collectively, our results offer novel insights into the neurobiological mechanisms underlying impulsivity and support the therapeutic potential of regionally targeted mGluR5 modulation for disorders characterized by dysregulated motor impulsivity.

## Introduction

Impulsivity is a key dimension in psychiatry, as elevated levels of impulsivity are associated with a variety of mental disorders, including attention-deficit hyperactivity disorder (ADHD) [1, 2], bipolar disorder [3], borderline personality disorder [4], schizophrenia [5] and addiction [6–8]. Defined as a tendency to act rapidly with little foresight of potential risks or negative consequences, impulsivity is a multifaceted construct encompassing both deficits in inhibitory control (motor impulsivity) and maladaptive decision-making (choice impulsivity), with the latter including delay discounting and risky decision-making [9–11]. Given its transdiagnostic relevance, the neurobiological underpinnings of impulsivity have been extensively studied both to better understand potentially shared pathophysiological processes across psychiatric disorders and to identify potential treatment targets that could benefit patients with a wide range of psychiatric conditions.

Extensive research has demonstrated that dopaminergic and serotonergic neurotransmission within the limbic cortico-striatal system plays a central role in regulating impulsivity [12–14]. However, despite these insights, effective treatments to control impulsivity remain limited [9], underscoring the need for further research into the neural underpinnings of impulsive behaviors. In recent years, accumulating evidence indicate that glutamatergic signaling also plays a pivotal role in impulsivity [15]. For instance, modulation of glutamatergic transmission through both N-methyl-D-aspartate (NMDA) and metabotropic glutamate receptors has been shown to influence impulsivity. Systemic administration of NMDA antagonists increases motor impulsivity [16, 17] and steepens delay-discounting [18–21] across a variety of tasks. Moreover, local infusions of NMDA antagonists into the medial prefrontal cortex (mPFC) enhance motor impulsivity [14, 22, 23], highlighting the role of glutamatergic cortical transmission in impulse control. Besides NMDA receptors, the metabotropic glutamate receptor subtype 5 (mGluR5) is gaining increasing attention, as accumulating evidence suggests its involvement in impulsivity. mGluR5 is highly expressed in the corticolimbic circuitry, where it is primarily localized postsynaptically on both glutamatergic and GABAergic neurons [24, 25]. mGluR5 plays an important role in regulating synaptic plasticity processes in these neurons [26–29] and is a key modulator of the excitatory/inhibitory (E/I) balance in the brain [30]. Furthermore, mGluR5 physically and functionally interacts with NMDA receptors [31], with reciprocal facilitation between their signaling pathways further contributing to synaptic plasticity [32, 33]. Alterations in mGluR5 expression have been consistently reported in several psychiatric disorders characterized by impulsive behaviors. For instance, genetic studies have linked an excess of copy number variations in GMR5, the gene encoding mGluR5, to ADHD [34, 35], and reduced GMR5 expression to major depression in males [36]. Reduced mGluR5 levels have also been reported, albeit inconsistently [37], in the prefrontal cortex (PFC) of patients with schizophrenia [38], bipolar disorder [38, 39] and major depression [40]. Furthermore, reduced mGluR5 availability has also been observed in individuals with cocaine [41–43], tobacco [44, 45], and alcohol use disorders [46, 47]. The most compelling evidence for a role of mGluR5 in impulsivity comes from preclinical studies showing that systemic administration of a mGluR5 positive allosteric modulator (PAM) decreases motor impulsivity [48, 49], while having no effect on delay-discounting [48]. In line with these findings, other studies have shown that mGluR5 antagonists do not alter delay-related [21] or risk-related choice impulsivity [50]. Collectively, these data suggest that mGluR5 may be specifically involved in motor impulsivity rather than in choice impulsivity, although more research is needed to confirm its specific role across impulsivity domains. Moreover, these studies relied on systemic administration of mGluR5 PAMs or antagonists, thus affecting multiple brain regions, and limiting the ability to identify the specific neural circuit or region that might be more directly involved. Interestingly, transcriptomic analysis focusing on the nucleus accumbens (NAc) have shown that GRM5 expression is downregulated in highly motor impulsive rats [51], suggesting that mGluR5 changes in the limbic circuitry may contribute to impulsive behavior. However, this study did not investigate GRM5 in other regions outside the NAc, and it is unknown whether this alteration is restricted to the NAc or extends to other brain regions.

Further research is thus needed to investigate potential links between mGluR5 expression and impulsive behavior, and to explore whether region-specific differences in mGluR5 availability may contribute to distinct impulsivity domains.

In the present study, we investigated mGluR5 regional brain availability in relation to two constructs of impulsivity, motor impulsivity and risky decision-making, using the Roman high- (RHA) and low-avoidance (RLA) rats. These rat lines exhibit distinct impulsivity profiles, making them a valuable model for studying the neurobiological substrates of impulsive behaviors. Compared to RLAs, RHAs display higher levels of motor impulsivity [52, 53], steeper delay-discounting, and also tend to exhibit greater risk-related impulsivity [54, 55], although the latter is less pronounced [56]. We assessed individual levels of both motor impulsivity and risky decision-making using the rat gambling task (rGT) and measured brain availability of mGluR5 in vivo using positron emission tomography (PET) and the specific mGluR5 antagonist radiotracer [^18^F]-PSS232 [57, 58]. Additionally, we used voxel-wise correlational analyses to identify potential brain regions where mGluR5 availability predicts these impulsive behaviors.

## Materials and methods

### Animals

Male RHA (n = 12) and RLA (n = 12) rats, aged three months, were used from our permanent colony of outbred Roman rats at the University of Geneva. Animals were housed three per cage and maintained under a 12-h-light-dark cycle (lights on at 7:00 a.m.), with controlled temperature (22 ± 2 °C) and humidity (50–70%). Rats were food-restricted and maintained at 85% of their free-feeding weight, whereas water was provided ad libitum. No randomization was used for allocation of animals to experimental groups, as the study involved two predefined rat lines (RHA and RLA). No blinding was performed during group allocation or outcome assessment. All experimental procedures were approved by the Animal Ethics Committee of the canton of Geneva and performed according to the Swiss Federal Law on Animal Care.

### Rat gambling task (rGT)

Rats were trained in operant chambers (Med Associates Inc., St Albans, VT, USA) with a house light, a wall containing five nose-poke holes (only the four outer ones were active), each equipped with a cue light and infrared sensors, and a food receptacle on the opposite wall dispensing 45 mg pellets (Test Diet ®, USA). Chambers were controlled via MedPC IV software. Rats were trained on the rGT as previously described [54, 55]. After two 30 min habituation sessions, they learned to nose-poke into illuminated holes for food rewards. Training continued with seven forced-choice sessions (one option per trial), followed by 25 daily 30-min test sessions where all four options were simultaneously available. Each trial began with a nose-poke into the illuminated pellet receptacle, followed by a 5 s inter-trial interval (ITI). Responding during this ITI was registered as a premature response, triggering a 5 s of time out (TO) and trial reset. After the ITI, the four apertures were illuminated, and the rat had 10 s to respond. Each option (P1–P4) differed in reward size, TO duration, and reinforcement probability: P1: p = 0.9, 1 pellet, and 5 s TO; P2: p = 0.8, 2 pellets, and 10 s TO; P3: p = 0.5, 3 pellets, and 30 s TO; P4: p = 0.4, 4 pellets, and 40 s TO. If the trial was punished, the hole’s cue light blinked at 0.5 Hz for the corresponding TO duration. No response within 10 s was recorded as an omission. P1 and P2 were considered optimal as they yielded more pellets with shorter time-outs, whereas P3 and P4 were non-optimal due to longer penalties and lower total rewards earned over the session. The percentage of choice for each option was calculated as [(number of choices for the option) / (number of total choices made)] × 100. Risk-related choice impulsivity was evaluated with the choice score [(%P1 + %P2) – (%P3 + %P4)]. The percentage of premature responses, used as an index of motor impulsivity, was assessed by the percentage of premature responses [(number of premature responses) / (total number of trials)] × 100. Other outcome measures were the percentage of omissions [(#omission responses / #total number of trials initiated) × 100], the choice latency, defined as the time between the end of the ITI and a nose-poke into a response hole, and the collect reward latency, defined as the time between reward delivery and a nose-poke into the food magazine. All behavioral measures were averaged across three consecutive sessions (with response variation <10% across all response holes).

### PET image acquisition and processing

[¹⁸F]-PSS232 was synthetized by the Center for Radiopharmaceutical Sciences, Swiss Federal Institute of Technology (Zurich), as previously described [58]. This radiotracer binds specifically to mGluR5, as evidenced by the lack of specific binding following mGluR5 blockade and in mGluR5 knockout rats and shows a test–retest variability of less than 10% [57, 58].

Animals were anesthetized with 2.5% isoflurane and implanted with a catheter in the tail vein. They were then positioned in a micro-PET scanner LabPET8 (Triumph II, TriFoil Imaging, Chatsworth, CA, USA), which has an in-plane resolution of 1.7 mm at the center of the field of view [59], using a custom-made stereotactic-like frame. A computer tomography (CT) scan was first performed for 5 min. Rats then received an i.v. injection of [^18^F]-PSS232 and were scanned for 45 min. The mean injected doses of [^18^F]-PSS232 did not significantly differ between rat lines (42.42 MBq ± 2.51 for RHAs, and 41.90 MBq ± 2.64 for RLAs; t = 0.14, p = 0.89), nor did the absolute mass dose (0.20 ± 0.10 µg per 100 g of body weight for RHAs and 0.19 ± 0.09 µg per 100 g of body weight for RLAs; t = 0.47, p = 0.64), nor the molar activity (21.97 GBq/µmol ± 33.56 for RHAs, and 21.05 GBq/µmol ± 30.93 for RLAs; t = 0.20, p = 0.84).

Dynamic PET images were acquired in list mode and reconstructed using the ordered subsets expectation maximization algorithm with 30 iterations. Image analysis was conducted using PMOD software (version 4.0, PMOD Technologies Ltd., Zurich, Switzerland). From the dynamic PET images, individual PET summation images were generated and checked for alignment with the corresponding CT images. Due to minor misalignment between the PET and CT images, all PET summation images were co-registered to their corresponding CT images using rigid registration based on normalized mutual information. CT images were then co-registered to the SIGMA magnetic resonance image (MRI) brain template [60] using mutual information-based rigid body registration. All these transformations were subsequently applied to the corresponding dynamic PET images, allowing the alignment of all rat brains to a common reference space. This led to coregistered PET-MRI images with an isotropic voxel size of 0.15 mm.

### PET data analyses

#### Region-of-interest analysis

A region-of-interest (ROI) template was defined on the SIGMA brain template [60]. ROIs were selected a priori based on the known distribution of mGluR5 availabilities in the brain [25], and included the ventral part of the mPFC - comprising the infralimbic (IL) and prelimbic (PL) subregions - the anterior cingulate cortex (ACC), dorsal striatum (dST), ventral striatum (vST), hippocampus (HI), amygdala (AM), thalamus (TH), and cerebellum. ROIs were defined as circular or elliptical regions positioned over the central plane of each structure to minimize partial voluming. The ROI template was applied to the MRI-coregistered dynamic PET images to produce regional time-activity curves. The non-displaceable binding potential (BP_ND_) of [^18^F]-PSS232 was estimated in each ROI using the Logan reference tissue method [61], with the cerebellum as reference, as previously described [57]. The optimal time t* for initiating the Logan linear fit was determined for each region and each subject using the PMOD modeling software. The algorithm incrementally increases t* from an initial value t* = 0 to the end of the scan, selecting the t* value at which further increases resulted in a maximum regression error of 10%, indicating a time stability of the resulting linear fit. Individuals t* values were typically below 11 min. The k₂′ was fixed at 5, based on Müller Herde et al. [57] who estimated this parameter using plasma input kinetic modeling of [¹⁸F]-PSS232 binding in the rat brain. BP_ND_ values from the left and right hemispheres were averaged to yield a single index of mGluR5 availability for each brain region.

#### Voxel-wise analyses

Voxel-wise parametric maps of [^18^F]-PSS232 BP_ND_ were calculated for each rat using the Logan reference tissue method and the cerebellum as reference. A [^18^F]-PSS232 BP_ND_ brain template of RHAs and RLAs was then developed as described previously [62]. Briefly, a representative [^18^F]-PSS232 BP_ND_ parametric scan was used as a reference. Parametric images of all rats were then normalized to the representative reference scan and averaged into a single scan. This averaged scan was duplicated and flipped from left to right, then subsequently averaged with its flipped duplicate to create a symmetrical voxel-wise averaged template. Finally, all parametric images were then spatially normalized to this template to correct for anatomical and positioning variability.

Parametric maps of [^18^F]-PSS232 BP_ND_ were then processed using Statistical Parametric Mapping version 12 (SPM12, Wellcome Trust Centre for Neuroimaging, London) as described previously [63]. Briefly, normalized BP_ND_ parametric images were scaled by a factor of 10 to establish a one-to-one relationship between the coordinates of the rat brain atlas of Paxinos & Watson [64] and the voxel size displayed in SPM12, which is designed on the actual size of the human brain. This approach allowed for minimal modifications to the default parameter settings in SPM12. Then, images were placed into a volume (“bounding box”) and smoothed with a Gaussian kernel of full width at half-maximum of 6 mm.

Voxel-wise statistical comparisons of [^18^F]-PSS232 BP_ND_ parametric images were conducted using unpaired two-sample t-tests to detect regional differences in mGluR5 availability between RHA and RLA rats. Moreover, SPM12 also provides the opportunity to identify voxels that covary with behavior without a priori assumptions. Whole-brain voxel-wise linear regression analyses were conducted to examine the relationship between [^18^F]-PSS232 BP_ND_ and both motor impulsivity and risky decision-making, identifying regions where mGluR5 availability is associated with each facet of impulsivity. The threshold of voxel‑level significance for group contrasts and regression analyses was set at p < 0.05, corrected for multiple comparisons using family-wise error (FWE) correction at the cluster-level. T-value maps of significant clusters were overlaid onto the MRI template, and anatomical localization was performed using the SIGMA template atlas [60].

#### Statistics

Normality of the data was assessed using a Kolmogorov-Smirnov test. Between-line differences in the percentage of premature responses and choice scores were tested using a two-tailed unpaired Student’s t-test. Before ANOVA analyses, homogeneity of variances was verified using Levene’s test. Sphericity was assessed using Mauchly’s test. Between-line differences in regional [^18^F]-PSS232 BP_ND_ were analyzed using a repeated measures ANOVA with rat line as a between-subject factor and brain region as a within-subject factor. Post-hoc comparisons were performed using Bonferroni’s test. Data were considered significant at p < 0.05 and are presented as mean ± SEM. Correlations between [^18^F]-PSS232 BP_ND_, as determined with the ROI approach, and the percentage of premature responses or choice score were tested using the Pearson’s correlation coefficient. No statistical methods were used to estimate sample size.

## Results

### Rat gambling task

**Fig. 1:**
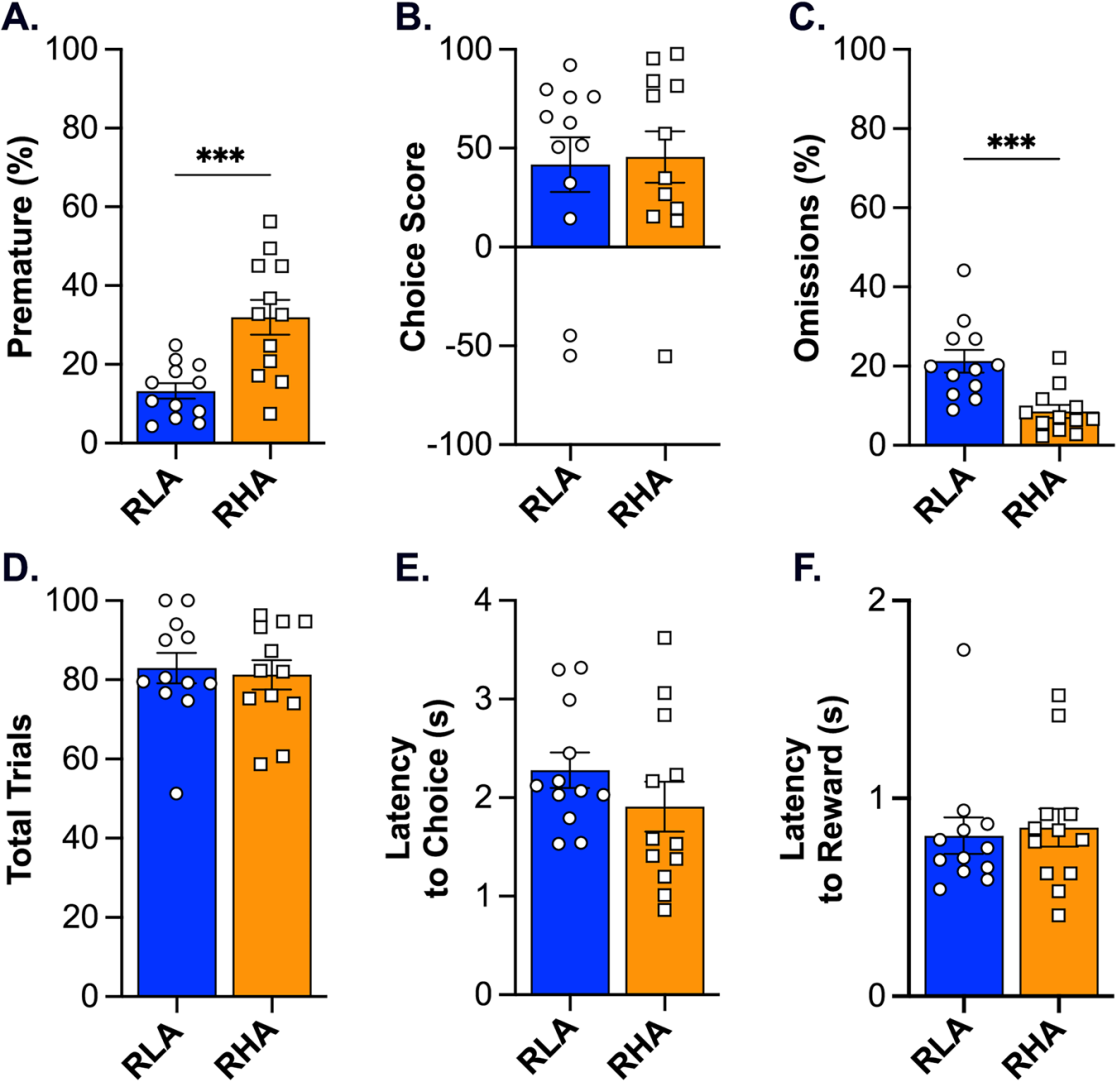
Assessment of motor and choice impulsivity in RHA and RLA rats on the rGT. Difference between RHA (n = 12) and RLA (n = 12) rats in: (**A**) percentage of premature responses, (**B**) choice score, (**C**) percentage of omissions, (**D**) totals number of trials, (**E**) latency to choice, and (**F**) latency to reward collection. For each parameter, values represent the average performance across three consecutive rGT sessions. Data are represented as mean ± SEM. Significantly different at *p < 0.05, **p < 0.01, and ***p < 0.001 using a two-tailed unpaired t-test.

In line with previous findings using the rGT [54, 55], RHA rats exhibited a significantly higher percentage of premature responses compared to RLA rats (t = 3.89, p < 0.001), indicating heightened motor impulsivity in RHAs (Fig. 1A). In contrast, no difference was observed in choice score between the two lines (t = 0.20, p = 0.84), indicating similar levels of risky decision-making in this animal cohort (Fig. 1B). The percentage of omissions was higher in RLAs (t = 3.87, p < 0.001; Fig. 1C) compared to RHAs, although both rat lines completed a comparable number of trials (t = 0.32, p = 0.75; Fig. 1D). There were no significant differences between RHAs and RLAs in choice latency (t = 1.97, p = 0.24 ; Fig. 1E) or reward collection latency (t = 0.30, p = 0.77 Fig. 1F).

### Regional brain differences in [^18^F]-PSS232 BP_ND_ between RHA and RLA rats

**Fig. 2:**
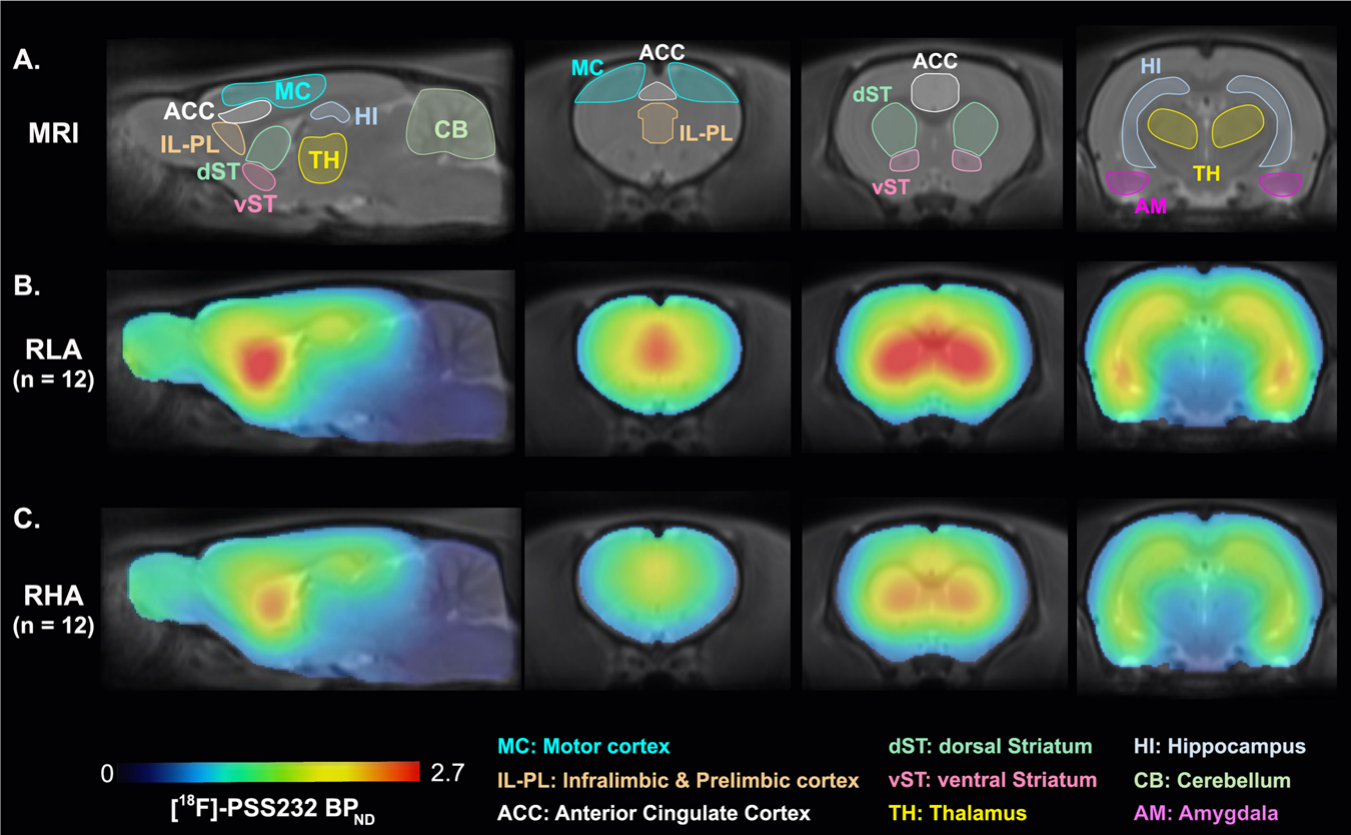
Brain distribution of [¹⁸F]-PSS232 BP_ND_ as an index of mGluR5 availability. (**A**) SIGMA magnetic resonance imaging (MRI) template of the rat brain showing the anatomical regions included in the region-of-interest analysis. (**B**) Average parametric maps of [¹⁸F]-PSS232 BP_ND_ for RLA rats (n = 12) and (**C**) for RHA rats (n = 12), superimposed onto the MRI template. Highest [¹⁸F]-PSS232 BP_ND_ values were observed in cortical regions, including the motor cortex (MC), infralimbic/prelimbic cortex (IL-PL), and anterior cingulate cortex (ACC), as well as in subcortical regions such as the dorsal (dST) and ventral (vST) striatum, thalamus (TH), amygdala (AM), and hippocampus (HI).

Figure 2 shows mean parametric maps comparing the distribution [^18^F]-PSS232 BP_ND_ in the brains of RHA and RLA rats. Both groups showed high mGluR5 availability in cortical regions, particularly in the ventral and dorsal parts of the mPFC - corresponding to the IL-PL region and the ACC, respectively - as well as in the striatum. Intermediate to low mGluR5 levels were observed in the AM, HI and TH. This regional distribution is consistent with previous studies using this radioligand in rats [57, 58] and aligns with ex vivo reports of mGluR5 brain distribution [25].

**Fig. 3:**
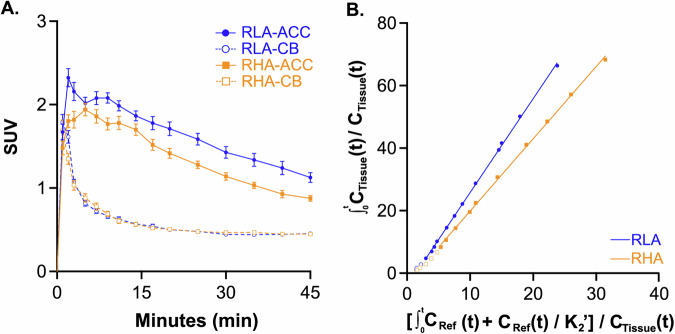
[¹⁸F]-PSS232 uptake and kinetic modeling in RHA and RLA rats. (**A**) Time-activity curves expressed as Standardized Uptake Values (SUVs) in the anterior cingulate cortex (ACC) and cerebellum (CB) for RHA and RLA rats. Data are shown as mean ± SEM. (**B**) Representative Logan reference tissue plots for one RHA and one RLA in the ACC.

Figure 3A compares mean time-activity curves (TACs) between RHA and RLA rats, obtained by ROI analysis in the cerebellum and the ACC, the latter serving as a representative target region. TACs in the cerebellum, used as the reference region, were similar between RHA and RLA rats, indicating comparable tracer delivery and non-specific binding in both rat lines. In contrast, TACs in the ACC were higher in RLA than in RHA rats, suggesting higher [^18^F]-PSS232 binding in this target region in the former rat line. This pattern supports the interpretation that the higher Logan-derived BP_ND_ observed in RLA rats reflects increased specific binding rather than differences in perfusion or early-phase kinetics. Logan plots showed excellent linearity in both lines, confirming the validity of the model (Fig. 3B).

**Fig. 4:**
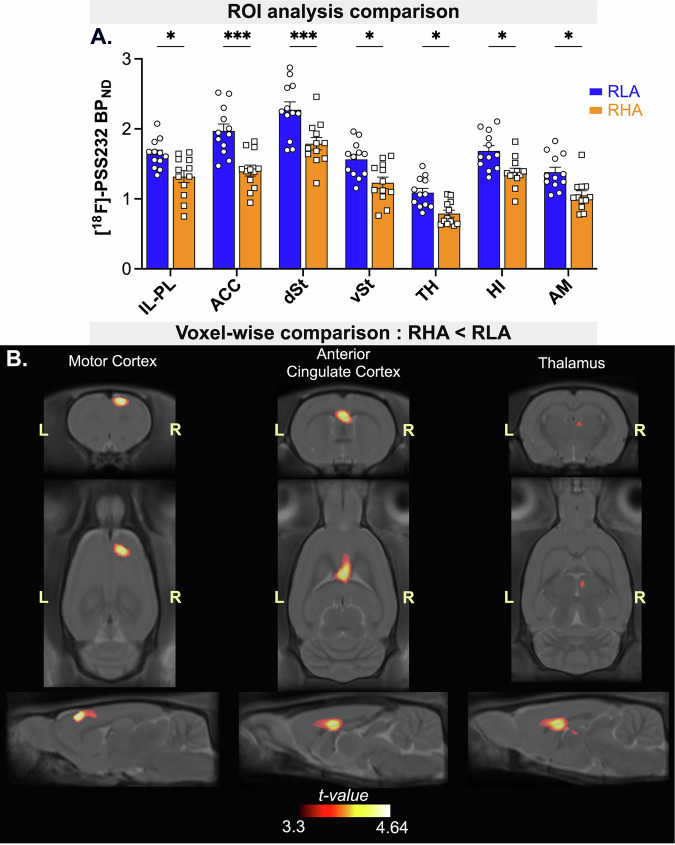
Regional differences in mGluR5 availability between impulsive RHAs and non-impulsive RLAs. (**A**) ROI-based analysis of [¹⁸F]-PSS232 images revealed widespread reductions in mGluR5 availability across all analyzed brain regions in RHA rats compared to RLAs, with the most pronounced deficits observed in cortical areas. Data are presented as mean ± SEM. Significantly different at *p < 0.05, **p < 0.01, ***p < 0.001 using a Bonferroni post-hoc test. (**B**) Voxel-wise t-statistical maps showing significantly lower [¹⁸F]-PSS232 BP_ND_ in RHAs compared to RLAs in the right MC (right: t = 4.64), ACC (t = 4.45), and right TH (t = 3.74). All maps are overlaid onto the SIGMA MRI rat brain template. The color scale represents t-values.

Using ROI analysis (Fig. 4A), a repeated-measures ANOVA revealed a main effect of rat line (F_1,22_ = 16.65, p < 0.001) and brain region (F_6,132_ = 115.6, p < 0.001) on [^18^F]-PSS232 BP_ND_, along with a significant interaction between the two factors (F_6,132_ = 2.67, p = 0.02). Bonferroni’s post-hoc comparisons indicated that RHA rats exhibited lower [^18^F]-PSS232 BP_ND_ in the IL-PL (p = 0.03), ACC (p < 0.001), dST (p < 0.001), vST (p = 0.02), TH (p < 0.05), HI (p = 0.04) and AM (p = 0.02) compared to RLA rats.

Figure 4B displays *t*-statistical maps of between-line differences in BP_ND_. Two clusters of significantly lower [^18^F]-PSS232 BP_ND_ were identified in RHA rats compared to RLA rats. One cluster was identified in the right hemisphere, overlapping the primary and secondary motor cortices (peak t = 4.64, p_FWE-corrected_ = 0.049; cluster size = 3.04 mm³). A second significant cluster (p_FWE-corrected_ = 0.012; cluster size = 4.85 mm³) encompassed two peaks: one in the ACC (peak t = 4.45) and the other in the right thalamus (peak t = 3.74). No cluster was identified with significantly higher [^18^F]-PSS232 BP_ND_ in RHA rats compared to RLA rats.

#### Relationships between [^18^F]-PSS232 BP_ND_ and impulsive behaviors

**Fig. 5:**
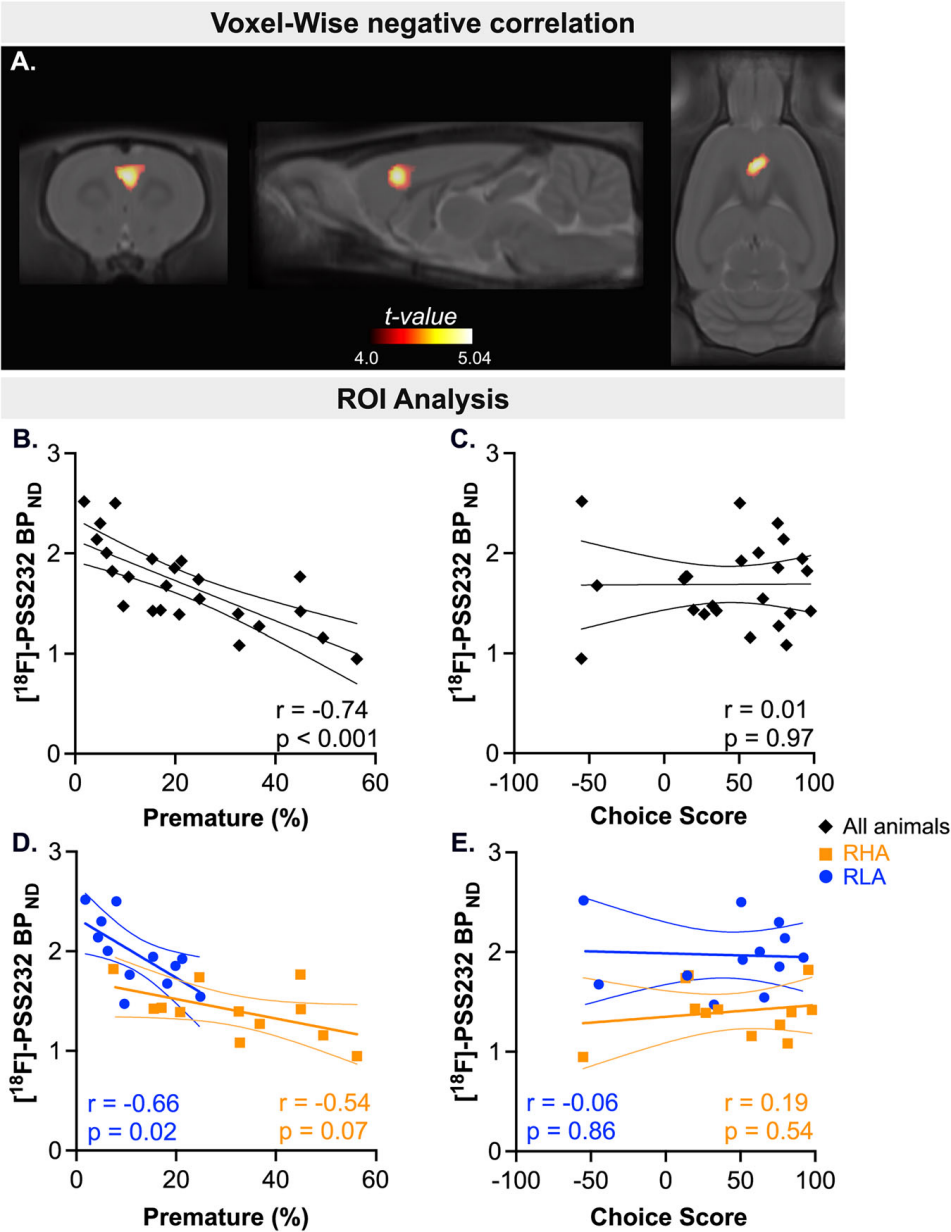
Negative correlation between mGluR5 availability in the ACC and motor impulsivity, but not risky decision-making. (**A**) Voxel-wise analysis revealed a cluster of significant negative correlation between [¹⁸F]-PSS232 BP_ND_ and the percentage of premature responses in the ACC (t = 5.04). The t-value color scale represents the strength of the negative correlation. (**B**) ROI-based correlations showing the relationships between [¹⁸F]-PSS232 BP_ND_ in the ACC and premature responses, and (**C**) choice score, based on pooled data from RHA and RLA rats. (**D**) Within-line ROI-based correlations showing the relationships between [¹⁸F]-PSS232 BP_ND_ in the ACC and premature responses, and (**E**) choice score.

A whole-brain voxel-wise regression analysis was performed to examine the relationship between [^18^F]-PSS232 BP_ND_ and impulsive behaviors. A significant cluster of negative correlation was found between premature responses and mGluR5 availability in the ACC (peak t = 5.04, p_FWR-corrected_ = 0.012, cluster size = 3.81 mm^3^; Fig. 5A), indicating that lower mGluR5 availability in the ACC is associated with higher levels of motor impulsivity. Notably, this cluster overlapped with the region where between-line differences in mGluR5 availability had previously been identified in the group contrast (Fig. 4B). In contrast, no significant clusters were found when examining positive correlations between motor impulsivity and mGluR5 availability. Similarly, no significant clusters of either positive or negative correlations were found between choice score and [^18^F]-PSS232 BP_ND_. These findings indicated that a strong association between reduced mGluR5 availability in the ACC and motor impulsivity, but not with risky decision-making. This result was further supported by ROI-based analysis, which indicated a significant negative correlation between premature responding and mGluR5 availability in the ACC (r = -0.74; p < 0.001; Fig. 5B). No significant correlation was observed between choice score and [^18^F]-PSS232 BP_ND_ in this region (r = 0.01; p = 0.97; Fig. 5C).

To verify that the relation between mGluR5 and motor impulsivity was not driven by between-group effects, we conducted within-line correlation analyses. In the ACC, premature responses were significantly negatively correlated with mGluR5 availability in RLAs (r = -0.66; p = 0.02; Fig. 5D) and showed a near-significant correlation in RHAs (r = -0.54; p = 0.07; Fig. 5D), supporting a link between ACC mGluR5 levels and motor impulsivity. No association between [^18^F]-PSS232 BP_ND_ and choice score was observed in either line (Fig. 5E).

**Fig. 6:**
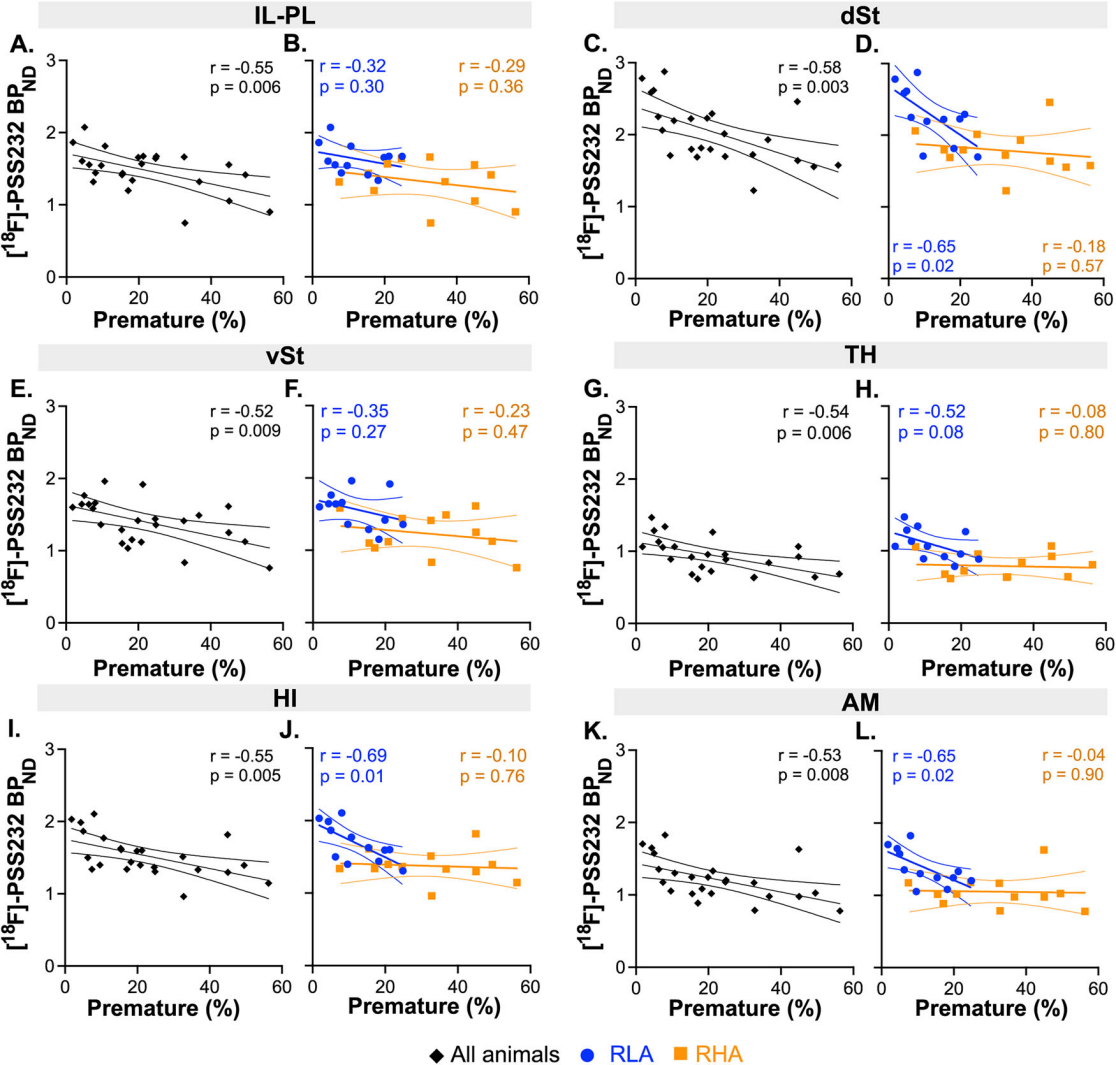
ROI-based correlations between mGluR5 availability and motor impulsivity. Panels **A**, **C**, **E**, **G**, **I**, and **K** show correlations across all animals, while panels **B**, **D**, **F**, **H**, **J**, and **L** display within-line correlations for RHA and RLA rats separately. Although significant negative correlations were observed when all animals were analyzed together, most of these associations did not remain significant when assessed within each line separately.

When evaluating the relationships between mGluR5 availability and impulsivity in other ROIs, and combining data from the two rat lines, significant negative correlations with premature responses were found in several regions (Fig. 6). However, most of these associations were no longer significant when examined within each line separately, except in the dSt (r = -0.65, p = 0.02), HI (r = -0.69, p = 0.01), and AM (r = -0.65, p = 0.02) where significant correlation persisted but in RLA rats only. No significant correlations were found between [¹⁸F]-PSS232 BP_ND_ and choice score, either when combining all animals or within each line separately (Fig. S1), suggesting no involvement of mGluR5 in this facet of impulsivity across any region. Additionally, no significant associations between [^18^F]-PSS232 BP_ND_ and omission rate were observed within any rat line or brain region (Fig. S2).

Finally, we conducted additional correlation analyses between [¹⁸F]-PSS232 BP_ND_ and premature responding, while statistically controlling for choice latency, reward collection latency, and omission rate as covariates, metrics known to reflect motivational and arousal states. Importantly, even after controlling for these variables, the voxel-wise correlation analysis still revealed a significant negative correlation between premature responding and mGluR5 availability, with the peak cluster located in the ACC (Fig. S3). Similarly, in the ROI-based analysis, the negative correlation between [¹⁸F]-PSS232 BP_ND_ and premature responding in the ACC remained significant after controlling for these variables (Table S1).

## Discussion

To our knowledge, this is the first in vivo study to investigate the relationship between regional brain mGluR5 availability and impulsivity, revealing a construct-specific association with potential translational relevance. While high motor impulsive animals exhibited widespread reductions in mGluR5 availability across multiple brain regions, voxel-wise analyses revealed that the most prominent differences between impulsive and non-impulsive individuals were confined within a functional network relevant to impulsive behavior and involving the motor cortex, ACC, and thalamus. Within this network, the ACC emerged as the brain region where mGluR5 deficits were most strongly associated with motor impulsivity, but not with risky decision-making. These findings align with previous evidence of mGluR5 dysregulation in psychiatric conditions characterized by impulsivity and highlight the ACC as a potential key node for mGluR5-mediated regulation of motor impulsivity. By identifying mGluR5 deficits in the ACC as a strong correlate of motor impulsivity, our results offer novel insight into the potential neurochemical mechanism that may underlie the elevated ACC glutamate levels that has been consistently linked to impulsivity in clinical populations. Moreover, these results underscore the therapeutic potential of regionally targeted mGluR5 modulation in disorders marked by impaired impulse control.

Our finding of an inverse relationship between mGluR5 availability in the ACC and motor impulsivity, but not risky decision-making, points to a specific involvement of mGluR5 in inhibitory control processes. This is further supported by prior studies showing that positive allosteric mGluR5 modulation reduces premature responding in the 5-CSRTT [48, 49]. In contrast, we found no evidence of an association between mGluR5 brain availability and risky decision-making, a facet of choice impulsivity. These results align with prior pharmacological findings showing that mGluR5 modulation, whether by PAMs or antagonists, does not alter performance in delay discounting [21, 48] or probability discounting [50] tasks. Altogether, these findings support the notion of a more specific involvement of mGluR5 in motor impulsivity, with limited involvement in choice impulsivity.

Impulsive behavior has commonly been linked to dysfunction within the PFC and its subcortical target, the basal ganglia (BG), with the cortico-BG-thalamo-cortical loop widely considered a central circuit underlying motor impulsivity [9]. In this framework, the ventromedial PFC, which includes the IL and PL cortices, and its projections to the NAc are considered key regulators of motor impulsivity, as lesion or inactivation of either of these regions in rodents reliably alter premature responding [9]. While this framework has been instrumental in advancing our understanding of impulse control, recent findings indicate that an additional circuit also plays a role in its regulation. Indeed, a cortico-thalamo-cortical loop involving projections from ventromedial thalamus (VM-TH) to the anterior lateral motor cortex (ALM), a higher-order motor area with premotor-like features primarily located in M1 with partial extension into M2 [65], has been identified as a critical substrate for motor impulsivity [66, 67]. Consistent with this emerging view, our findings show that rats with high motor impulsivity exhibit most pronounced reductions in mGluR5 availability in the mediodorsal thalamus (MD-TH), in the ACC, and in a region overlapping M1 and M2 that is anatomically consistent with the ALM. Although projections from the MD-TH to the ALM are weaker than those from the VM-TH [68] and have not yet been studied for their potential role in modulating impulsivity, they may possibly also contribute to thalamo–cortical signaling relevant to motor control. Indeed, the MD-TH exhibits reciprocal connections with the ACC [69, 70], a region implicated in anticipatory control [71], error monitoring [72, 73], and adaptive motor control [74]. Notably, MD-TH can modulate ACC activity via feedforward inhibition mediated by parvalbumin interneurons [70], suggesting a potential mechanism through which MD-TH mGluR5 deficits might influence motor impulsivity. The ACC also projects to M1 and M2 [69, 75, 76], which are central to movement preparation and execution [77]. Together, these regions are integral of a functional cortico-thalamo-cortical network increasingly recognized as essential for motor planning and action selection [78]. Cortico-thalamo-cortical circuits have been proposed to function synergistically with the canonical cortico-BG-thalamo-cortical circuit, potentially under BG gating, to facilitate anticipatory motor control and regulate motor impulsivity [67, 79]. Our data support this model and further suggest that disruptions in mGluR5 signaling within these parallel circuits may compromise the signal integration necessary for effective behavioral inhibition, thereby contributing to the expression of motor impulsivity.

Strikingly, although mGluR5 deficits were found across several brain regions in motor impulsive individuals, suggesting broad alteration in glutamatergic signaling, voxel-wise analysis identified the ACC as the brain region where mGluR5 deficits were most strongly associated with motor impulsivity, but not with risky decision-making. Supporting this, ROI analysis indicated that only the ACC showed a robust within-line association between mGluR5 availability and premature responses in low-impulsive RLAs, along with a near-significant relationship (p = 0.07) in high-impulsive RHAs. No other brain region showed comparable within-line correlations across both phenotypes. Indeed, although other regions displayed correlations within RLA rats, these effects were not observed in RHAs and therefore lacked cross-phenotype consistency, suggesting that mGluR5 deficits in those regions may reflect, at least in part, other phenotypical differences between the two rat lines, such as differences in novelty seeking or anxiety-related behavior [80]. The ACC was the only region showing convergent within-line effects across both rat lines, with weaker but directionally consistent evidence in RHA rats, and thus emerged as the region most consistently linking mGluR5 availability to motor impulsivity. This finding is in line with prior evidence linking the ACC to impulsive behavior. For instance, chemogenetic inhibition of glutamatergic pyramidal neurons [81] or chemo-activation of parvalbumin-expressing GABAergic neurons in the ACC [82] both lead to a reduction in premature responding in rodents. Moreover, chemo-inhibition of ACC neurons projecting to the TH decrease premature responding [83], further substantiating a role of this pathway in promoting motor impulsivity. Complementing this, human neuroimaging studies showed that ACC activity correlates positively with false alarms, a proxy for impulsive motor responses, during a response inhibition task [84, 85]. Collectively, these studies position the ACC as a key hub in the neural regulation of motor impulsivity and suggest that hyperactivity or dysregulation in the ACC contributes to heightened motor impulsivity. Supporting this, clinical studies in humans have consistently shown that impulsivity, as assessed with the Barratt Impulsiveness Scale or the Go/No-Go task, is positively associated with glutamate levels and negatively associated with GABA levels in the ACC in both healthy individuals [86] and in patients with conditions characterized by impaired impulse control, including ADHD, bipolar disorder, and opioid use disorder [87–89], suggesting that a disrupted E/I balance in the ACC may underlie impulsivity. While the mechanisms driving this imbalance is unknown, our data showing an association between mGluR5 deficits in the ACC and motor impulsivity point to impaired mGluR5 signaling as a potential contributor. Indeed, these receptors are expressed on both excitatory pyramidal neurons and inhibitory GABAergic neurons [24, 90], where their activation enhances neuronal excitability [28]. In cortical circuits, mGluR5 activation on GABAergic neurons enhances their intrinsic excitability, thereby promoting inhibitory control over pyramidal neurons and contributing to the maintenance of E/I balance [30]. In parallel, mGluR5 expressed in cortical excitatory neurons influences the development of inhibitory circuits, as its deletion leads to reduced inhibitory inputs onto principal excitatory neurons, indicating a non-cell-autonomous role in shaping GABAergic network formation [91]. Similarly, selective deletion of mGluR5 from GABAergic interneurons also disrupts the normal development of inhibitory circuits [92], further underscoring the importance of mGluR5 signaling in establishing balanced cortical connectivity. Together, these findings suggest that innate deficits in cortical mGluR5, and notably in the ACC, may primarily impact GABAergic signaling, leading to disinhibition of pyramidal neurons, heightened glutamatergic output, and ultimately, increased impulsive behavior. This hypothesis is supported by findings that activating GABA interneurons in the ACC reduces premature responding [82], suggesting that restoring inhibitory tone within the ACC contribute to decrease motor impulsivity.

Interestingly, several studies have shown that positive allosteric modulation of mGluR5 reduces premature responding in the 5-CSRTT [48, 49]. This finding that enhancing mGluR5 signaling reduces motor impulsivity is consistent with our data linking mGluR5 deficits to this specific facet of impulsivity. As previously discussed, these compounds may contribute to restore cortical E/I imbalance disrupted by reduced mGluR5 availability. Supporting this view, mGluR5 PAMs have been shown to counteract the pro-impulsive effect of the NMDA antagonist MK801 in the 5-CSRTT [48]. Additionally, they also simultaneously reverse MK801-induced reduction in GABAergic neuron firing and elevation in glutamatergic neuron firing [93], and prevent MK801-induced elevation in glutamate levels in the mPFC [94], suggesting a normalization of cortical function. Together, these findings suggest that mGluR5 PAMs may exert anti-impulsive effect, at least in part, by restoring inhibitory control and stabilizing glutamatergic tone in prefrontal circuits.

This study presents several limitations that warrant consideration. First, the exclusive use of male rats limits the generalizability of our findings across sexes. This choice was made to minimize potential confounding effects of hormonal fluctuations in females, as motor impulsivity has been shown to vary across the estrous cycle [95], and sex differences have been reported in both decision-making [96] and motor impulsivity [97]. Additionally, sex differences in mGluR5 level have also been reported in mice [98], although findings in humans did not found such differences [99]. Future studies should therefore investigate whether the association between mGluR5 availability in the ACC and motor impulsivity extends to female rats. Secondly, as RHAs and RLAs are inbred lines, the mechanisms underlying impulsivity in this model may not fully reflect the complex, polygenic architecture of motor impulsivity in humans. While our findings provide an important initial insight, future research using more genetically diverse animal models and human populations will be essential to evaluate the broader translational relevance of our results. Finally, given the correlational nature of our study, the observed association between mGluR5 deficits and motor impulsivity does not establish a causal role for mGluR5 dysfunction in impulsivity.

In conclusion, this study provides the first in vivo evidence that reduced mGluR5 availability in the ACC is associated with elevated motor impulsivity, but not with risky decision-making. The relative regional specificity of these deficits and its association with a particular facet of impulsivity offer novel insight into the neurobiological mechanisms underlying impulse control. Reduced mGluR5 availability may disrupt local glutamatergic transmission, potentially contributing to dysregulated excitatory activity within cortical circuits implicated in inhibitory control. While the current findings are correlational, they provide an initial framework for future research aimed at establishing a causal link between mGluR5 function and impulsive behavior. Such studies should investigate the cell-type specificity and functional consequences of mGluR5 deletion, especially in the ACC, as well as whether targeted enhancement of mGluR5 signaling in this region can restore inhibitory control in individuals with heightened motor impulsivity.

## Supplementary information

Supplementary results

## Data Availability

Source data have been deposited in the Zenodo database under accession code: 10.5281/zenodo.16411919.
