# Supplementary material for: Metabotropic glutamate receptor 5 in the anterior cingulate cortex predicts individual differences in motor impulsivity but not in risky decision-making
Source: Transl Psychiatry. 2026 Mar 23;16:192. doi: 10.1038/s41398-026-03951-5 (PMC13039440; doi:10.1038/s41398-026-03951-5)
Supplement: Supplementary file 1 — Supplementary results [file 41398_2026_3951_MOESM1_ESM.docx]

**Supplementary results**

**
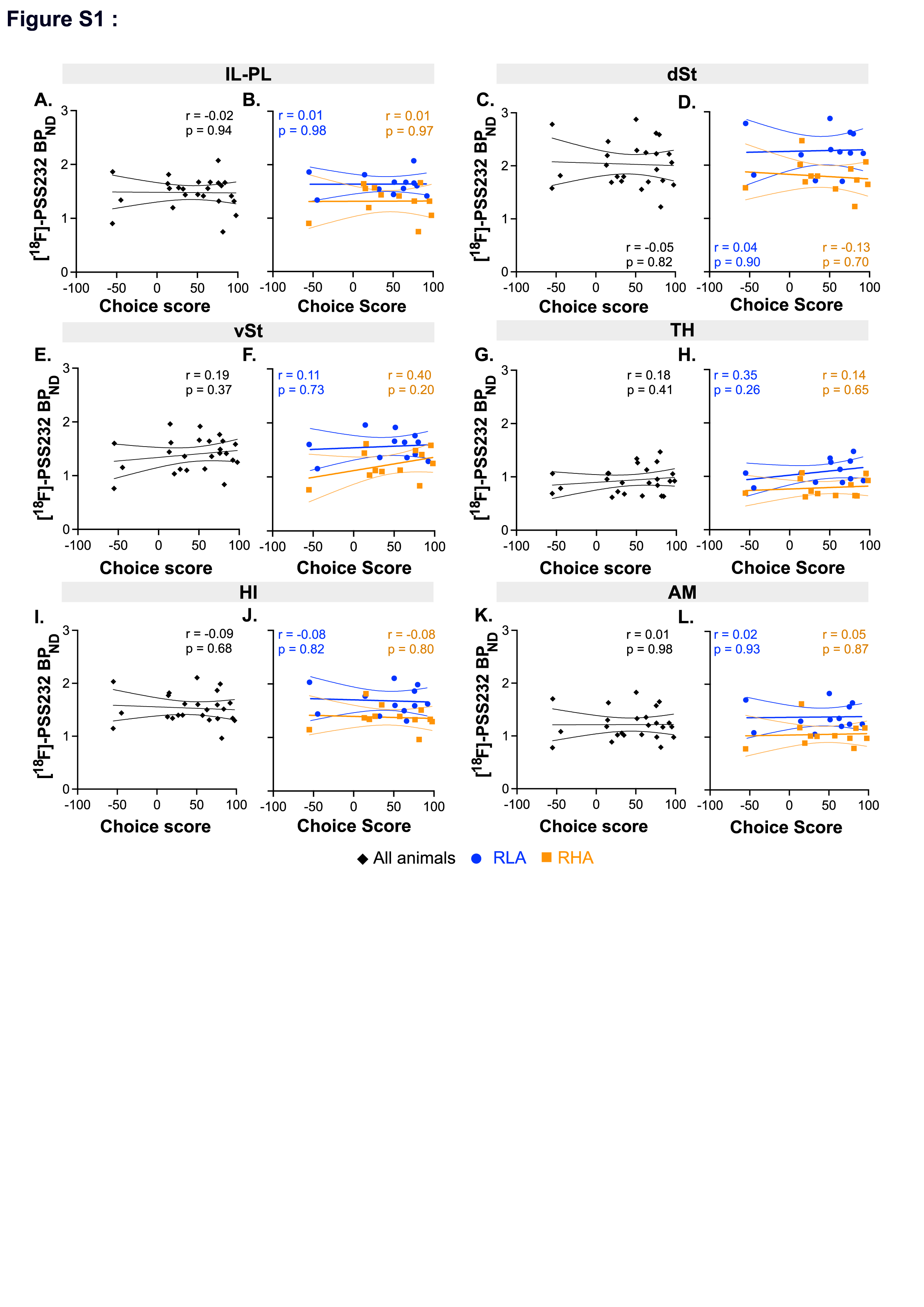
**

**Figure S1: ROI-based correlations between mGluR5 availability and risky-decision making.** Panels A, C, E, G, I, and K show correlations across all animals, while panels B, D, F, H, J, and L display within-line correlations for RHA and RLA rats separately. No significant correlations were observed between mGluR5 availability and risky decision-making in any of the ROIs investigated, whether the data were pooled across both lines or assessed separately within each line.

**
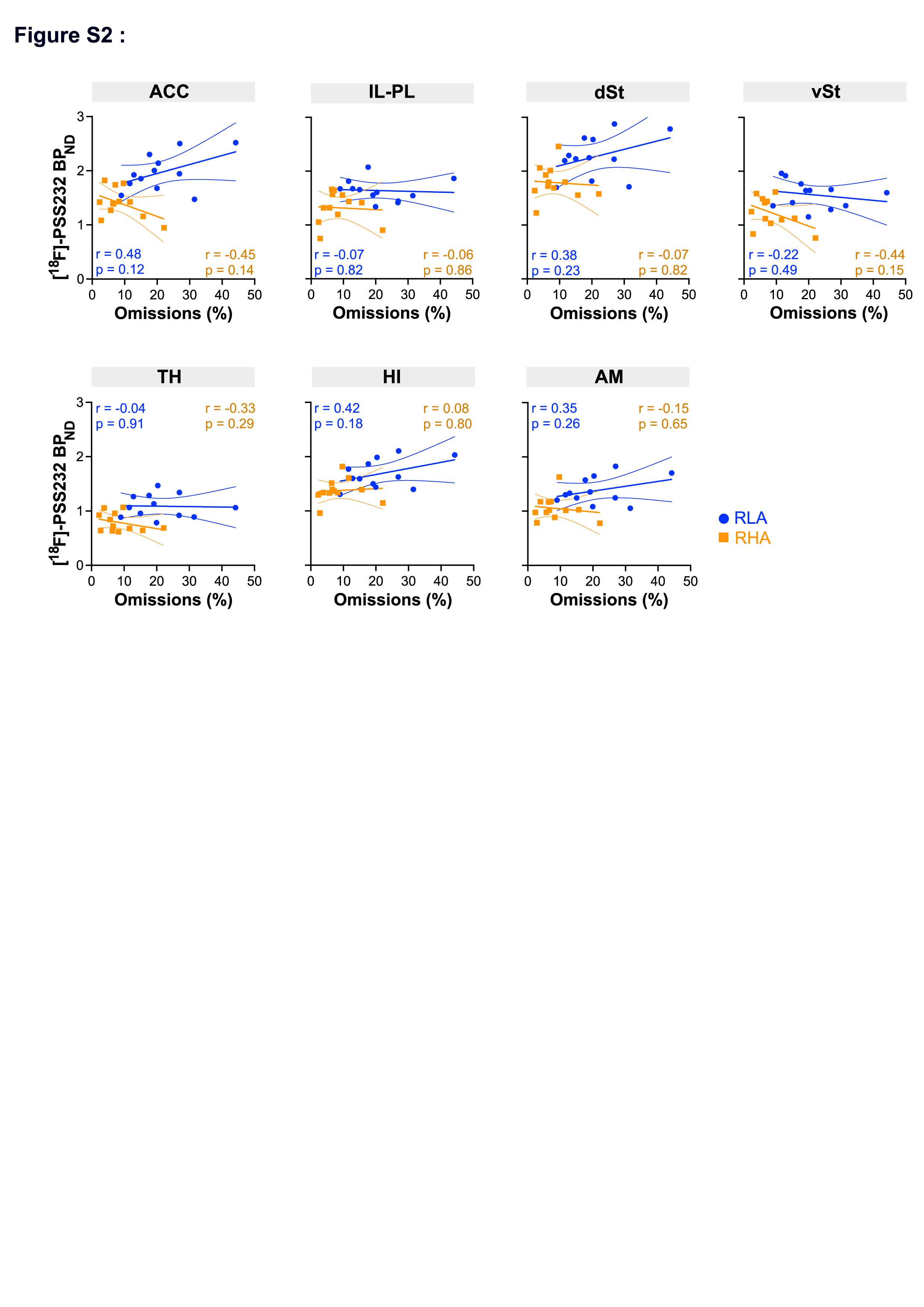
**

**Figure S2: ROI-based correlations between mGluR5 availability and percentage of omissions.** No significant associations were observed in any brain region within either line.

**
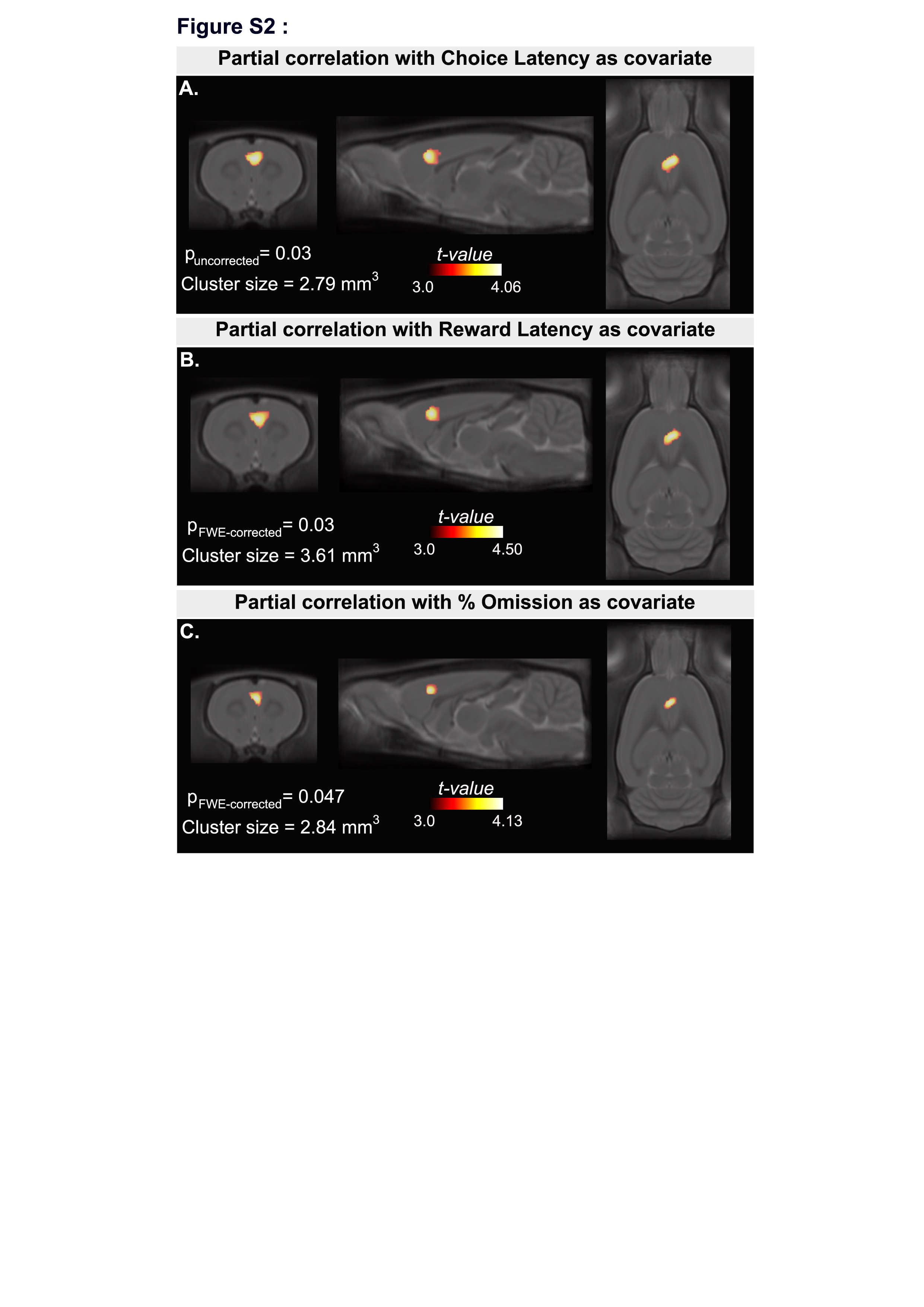
**

**Figure S3: Voxel-wise correlation between mGluR5 availability and premature responding, controlling for motivational variables**. The cluster of negative correlation between premature responding and [¹⁸F]-PSS232 BP_ND_ remained significant in the ACC after controlling for (A) choice latency (*t* = 4.06), (B) reward collection latency (*t* = 4.50), or (C) percentage of omissions (*t* = 4.13), indicating that this association is not driven by motivational confounds, but rather reflects a specific link to motor impulsivity.

**Table S1: ROI-based partial correlations between [^18^F]-PSS232 BP_ND_ in the ACC and motor impulsivity, controlling for choice latency, reward collection latency or percentage of omissions.**

| Covariates | Choice Latency | Reward Latency | Omissions (%) |
| --- | --- | --- | --- |
| Pearson’s r | -0.64 | -0.69 | -0.62 |
| p-value | < 0.001 | < 0.001 | 0.002 |
